# Oxide and Hydrogel Inverse Opals and Their Applications as Physical, Chemical and Biological Sensors

**DOI:** 10.3390/s25113370

**Published:** 2025-05-27

**Authors:** Peter Hutchison, Peter Kingshott, Aimin Yu

**Affiliations:** Department of Chemistry and Biotechnology, Swinburne University of Technology, Hawthorn, VIC 3122, Australia; phutchison@swin.edu.au (P.H.);

**Keywords:** inverse opals, sensors, photonic colloidal crystals

## Abstract

Inverse opal (IO) structures based on photonic colloidal crystal (PCC) templates are types of materials that possess unique optical properties due to their ordered arrays. These materials have the ability to manipulate the propagation of light, producing unique reflection spectra and structural colours. Due to these properties, IOs have been used as optical sensors for various applications such as the detection of physical, chemical, and biological entities. This review begins with a brief introduction of PCCs, IOs and their preparation procedures. The recent advancements in the applications of IOs for sensing temperature, pH, humidity, chemical compounds (such as organic solvents and heavy metal ions), and biological entities (such as tumour cells, viruses and bacteria) are then discussed in detail. The review also explores strategies and techniques aimed at enhancing the sensitivity and lowering the limit of detection of IO-based sensors. Finally, it addresses the current challenges, existing limitations, and prospective future directions in the development and deployment of IO-based sensors.

## 1. Introduction

At present, there are numerous analytical methods for the detection of physical, chemical, and biological entities such as chromatography, electrochemistry, fluorescence, Raman spectroscopy, and enzyme-linked Immunosorbent Assay (ELISA). These methods and instruments however have various problems associated with them such as their size and the need of trained personnel to operate them within a laboratory. A faster, more compact, and user-friendly method is therefore required. A solution to these problems which has attracted a large amount of interest and research over the last few decades is the use of optical sensors, or more specifically photonic colloidal crystal sensors. These sensors rely upon the manipulation of light due to their crystalline nature and consequently undergo a colour change in response to physical, chemical and biological stimuli.

A crystal is a periodic arrangement of atoms or molecules. The pattern of which atoms or molecules are arranged and repeated in space is known as the crystal lattice. The smallest representation of a crystal is the unit cell. All crystal lattices are built of repeating unit cells, and most naturally occurring and artificially prepared solids are crystalline. A specific type of crystal composed of colloidal particles, known as photonic colloidal crystals (PCCs), was first developed in 1987 by Yablonovitch and John based upon the properties of semiconductor crystals [1]. The most common assembly of colloidal particles is the hexagonal packing arrangement. Due to their spatial periodicity, these materials can strongly modulate electromagnetic waves [2] and therefore have the capacity to control the propagation of light according to the specific constituents of the crystal and the geometry of the lattice.

One of the most important optical properties of a PCC is the existence of a phenomenon known as a photonic band gap (PBG), which prohibits the propagation of light waves of specific frequencies within the structure of the crystal. As light waves within the region of the PBG cannot travel through the material, they are consequently reflected. When the reflected light waves are within the visible range (350–700 nm), a bright colour known as the ‘structural colour’ is reflected by the photonic crystal. This phenomenon occurs within nature, producing the colours upon a beetle, the feathers of a peacock, the wings of a butterfly [3], and within the colours of the opal gemstone. The light waves with frequencies outside the PBG, however, travel through the material and are consequently not reflected. Due to the PBG phenomenon, various applications exist within the field of optical fibres, smart devices, and various types of sensors [4].

**Figure 1:**
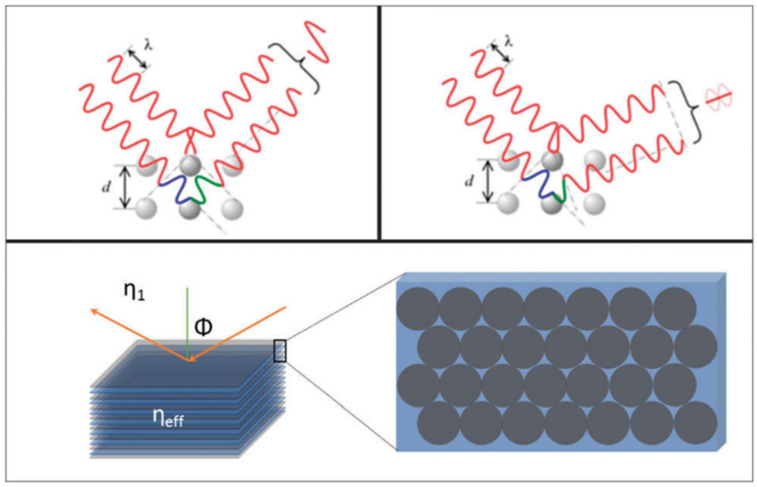
Schematic diagram demonstrating the interaction of light with the periodic material with an effective refractive index, and the light scattered by the sphere plains [5]. Copyright 2015, Royal Society of Chemistry, London, UK.

The PBG of a photonic colloidal crystal follows the angle-dependent diffraction of colour and can be expressed generally as the Bragg’s-Snell law (a combination of Bragg’s law and Snell’s law):

(1) $$m\lambda_{m}=2d\sqrt{\eta_{eff}-\sin^{2}\phi_{1}}$$

where: *η*_eff_ is the effective refractive index of the media, and depends on the relative refractive indices of spheres, surrounding medium, and the packing density. *m* is the diffraction order, *λ*_m_ is the wavelength of the reflected light, *d* is the interplanar spacing and *ϕ* is the incident light angle [5]. Figure 1 demonstrates this phenomenon.

**Figure 2:**
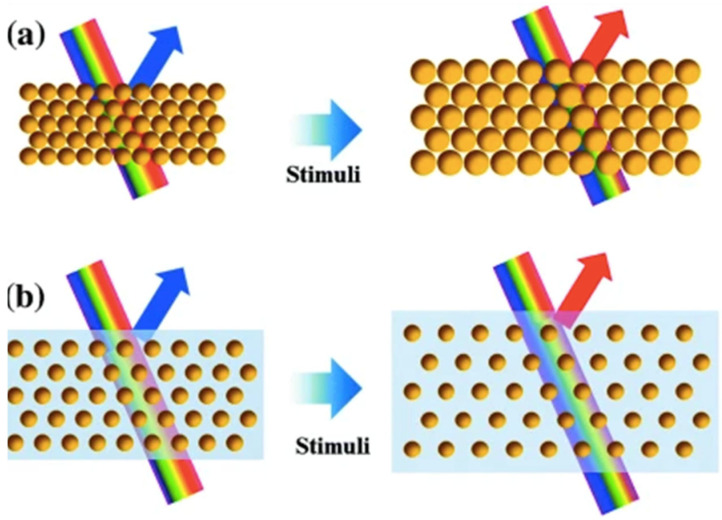
A schematic diagram representing the effect of (**a**) the size of particles, and (**b**) the spacing between particles upon the PBG when light interacts with a PCC. Reprinted from Ref. [6]. Copyright 2016, Springer, Berlin/Heidelberg, Germany.

Due to this principle, when the spacing between the planes (*d*) of a PCC changes, the diffracted wavelength will change accordingly (producing different colours). If the spaces between the particles become closer, a reflection towards the blue end of the spectrum will occur (known as a blue-shift), and conversely, if the space between the particles becomes greater, a reflection towards the red end of the spectrum will occur (known as a red-shift). A colour change will also occur due to the size of the particles. If the size of the particles becomes larger, a red-shift will occur, and if the particles become smaller, a blue-shift will occur, as shown in Figure 2 [6].

The idea of utilising the PBG properties of PCCs was first proposed by Yablonovich and John in 1987. Yablonovich discovered that PCCs had a region within them where spontaneous light emission could be controlled by preventing the propagation of light, and consequently came up with the term and theory of the PBG [7]. Since Yablonovitch’s discovery of the PBG, research into PCCs has been developed to cover the spectral regions from microwaves to ultraviolet frequencies [8]. Various and numerous applications can therefore be employed to utilise this phenomenon. One of the main applications being sensors, where a structural colour change can be either observed with the naked eye, or the PBG change measured with an instrument, when a PCC material is exposed to certain external stimuli such as temperature, pH, humidity, mechanical deformation, or the presence of ions, molecules, and various biomolecules.

## 2. Inverse Opal Structures via Templating Photonic Colloidal Crystals

### 2.1. Methods for the Preparation of PCCs

**Figure 3:**
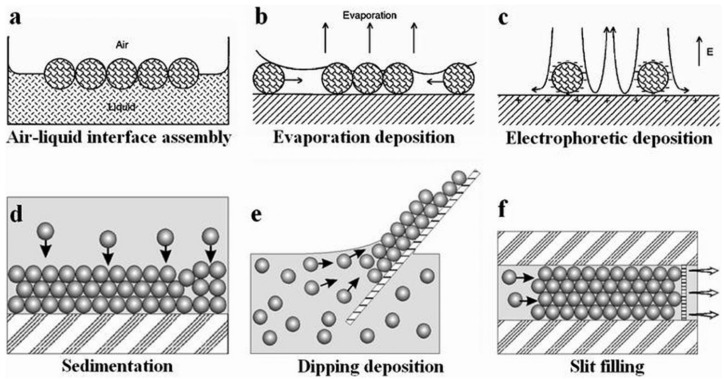
Illustration of different methods used for the assembling of 2D and 3D colloidal crystals. (**a**) air-liquid interface assembly (**b**) Evaporation deposition (**c**) Electrophoretic deposition (**d**) Sedimentation (**e**) Dipping deposition (**f**) Slit filling. Reprinted from Ref. [12]. Copyright 2013, Royal Society of Chemistry, London, UK.

Nature is a master at self-assembling hierarchically structured multifunctional materials [9]. Self-assembly is a process whereby individual components arrange themselves into an ordered structure [10]. Within the field of science, self-assembly has been inspired and mimicked from natural processes and systems within the laboratory. Self-assembly occurs due to various forces between colloid particles. In the case of colloidal spheres, forces such as electrostatic interactions, hydrogen bonding interactions, hydrophilic/hydrophobic effects, charge compensation, and capillary action all influence the self-assembly process [11]. A wide variety of self-assembly methods exist. The following sections outline some of the most commonly used methods for the synthesis of PCC structures. Figure 3 illustrates six of these methods [12].

### 2.2. Methods for the Preparation of Inverse Opal

**Figure 4:**
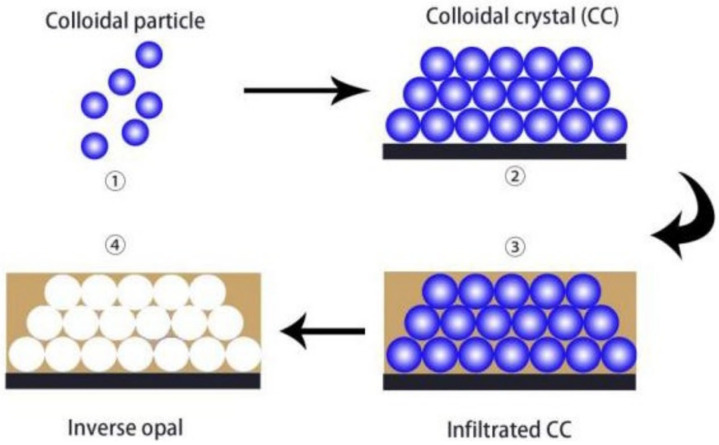
Schematic representation of the general preparation procedure of IO via templating PCC. Reprinted from Ref. [13]. Copyright 2023, MDPI, Basel, Switzerland.

One of the most useful structures produced from PCC assemblies are inverse opal photonic crystals (IOPC) structures, or more generally Inverse opal (IO). IO structures are highly porous structures with interconnected air pores that are arranged in a close-packed array. Similar to PCCs, they also have the ability to undergo a colour change when the pore sizes are within the scale of visible light, due to the PBG of the material. IO structures can be achieved by templating colloidal crystals. This involves filling the spaces between the spheres of a colloidal crystal assembly with a fluid material such as a polymer, a silica-based material such as tetraethyl orthosilicate (TEOS), or various metal oxides. After the fluid has been converted into an interconnected solid skeleton, the microsphere templates are then removed by calcination or chemical etching to acquire the inverse opal structure. Figure 4 demonstrates this process [13].

### 2.3. Common Photonic Crystals for Sensing

Silica-based, metal oxide, and hydrogels are the most common materials used for sensing purposes. Based on the sensing mechanism, various types of sensors have been reported including physically responsive sensors (such as temperature, pH, humidity, mechanical force, electric field, magnetic field, etc.), chemically stimuli-responsive sensors (such as volatile organic compounds, organic gases, heavy metal ions, etc.), and biologically stimuli-responsive sensors (such as nucleic acids, enzymes, antibodies, biomacromolecules, etc.) [3].

Silica-based and metal oxide IO, commonly consisting of SiO_2_, TiO_2_, ZrO_2_, ZnO_2_, can be used for the detection and presence of various chemical compounds including organic solvents, heavy metal ions, organic gases, and toxic organic pollutants. To detect these compounds, the sensing mechanism usually relies upon a change within the refractive index before and after the material fills the pores of the IO. When this occurs, a PBG, or structural colour change takes place, which can be observed either visually or measured with an instrument such as UV/Vis or an ellipsometer. As different silica-based and metal oxide IO possess different refractive indexes, and different sized pores within the IO produce different colours, these two properties can be utilised to design a specific sensing mechanism. Biological entities such as cells, viruses, amino acids, and bacteria can also be detected by silica and metal oxide IO. This usually involves functionalising the pores within the IO material surface with aptamers, antibodies, or other cross-linkers to immobilise the desired entity, then measuring a change within the PBG due to refractive index changes.

**Figure 5:**
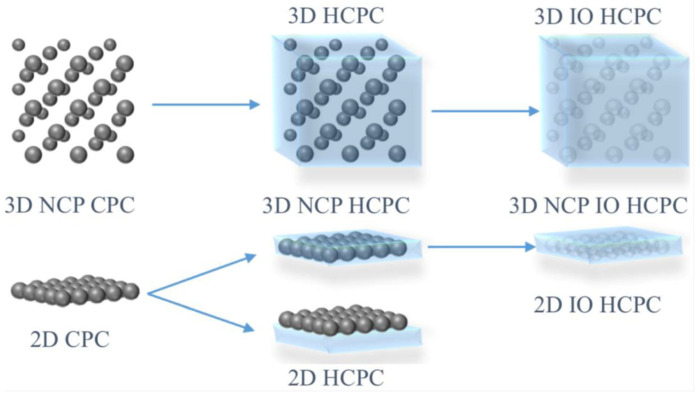
Schematic diagrams for the preparation of 3D non-close-packed (NCP) IO Hydrogel-based colloidal photonic crystals (HCPC) otherwise known as photonic colloidal crystal hydrogel (PCCH), and 2D IO HCPC. Reprinted from Ref. [1]. Copyright 2020, MDPI, Basel, Switzerland.

Hydrogels, usually consisting of various acrylamides such as the thermosensitive polymer poly(N-isopropylacrylamide) (PNIPAM) [14] or 2-hydroxyethyl methacrylate-co-acrylic acid (HEMA-AA) [15,16,17] are common materials used to produce an inverse opal structure known as hydrogel inverse opal (HIO). Hydrogels are three-dimensional water-insoluble networks, formed by physical or chemical methods by polymers containing large amounts of a water mobile phase [1]. The construction of a HIO involves a process where a PCC template (both non-close packed and close-packed) is infiltrated with an acrylamide then treated with UV polymerisation. The PCC template is then removed by chemical etching to produce hydrogel inverse opal (HIO), as illustrated in Figure 5.

HIOs have the capacity to absorb and retain large amounts of water (60–90 wt.%) within their porous structure [18] resulting in the swelling of the hydrogel material, known as volume phase transition (VPT). The VPT can occur when HIO is exposed to various external stimuli. For instance, thermosensitive HIO swells or shrinks when exposed to a change in temperature, altering the periodic structure of the crystal lattice. As the swelling and shrinking changes the conformation of the pores, a change in the PBG occurs, resulting in a structural colour change. HIO materials have a wide field of applications for the detection of physical properties due to their capacity to shrink or swell in response to various physical conditions. It can therefore be used as a sensor for the detection of physical properties such as temperature, humidity, pressure, and mechanical force.

Another type of PCC sensor which is not an inverse opal structure combines a PCC template within a hydrophilic polymer [19]. This combined material, known as a photonic crystal hydrogel (PCH), once again has the ability to swell or shrink in response to specific external stimuli, due to the alteration of the periodic arrangement of the PCC template within the hydrogel. For instance, Ma et al. [19] produced a PCH using phenylboronic acid (PBA) derivatives and cis-diols. The prepared microgels with PBA groups were placed within a glycomonomer solution to assemble into ordered colloidal crystals. Due to the ability of PBA to perform reversible interactions with the *cis*-diol groups of glycomonomers, dynamic crosslinking of adjacent microgel particles through reversible PBA-*cis*-diol interactions between the microgels and the resultant glycopolymers was achieved [19]. PCHs therefore have various applications as a sensor also due to their ability to swell or shrink in response to various chemicals. This material often contains specific linkers such as aptamers between the dispersed colloidal particles which have the ability to shrink when they encounter entities such as heavy metals. This conformation change then produces a PBG, or structural colour change.

## 3. Applications of HIO and PCCHs in Sensing

### 3.1. IO and PCC Hydrogels as Physically Responsive Sensors

Hydrogels are the main type of materials used for the detection of physical changes due to their ability to swell or shrink in response to changes within a physical environment such as alterations in temperature, pH, humidity, mechanical force, and environmental pressure. Silica or metal oxide-based IO is generally not used for this purpose as the material has a solid ridged structure which generally cannot swell or shrink and therefore respond to the above-mentioned changes within physical environments.

#### 3.1.1. Detection of Temperature

The detection of temperature is often important within various industries. It is therefore necessary to monitor any temperature changes in an easily observed manner. Various methods of producing such sensors have been studied and investigated using a wide number of different materials [20,21,22,23,24]. One of the main materials used for this purpose are polymer-based hydrogels. For instance, studies carried out by Takeoka et al. [23] prepared inverse opal-structured thermosensitive hydrogels from *N*-isopropylacrylamide (NIPA). Due to NIPA’s capacity to undergo thermo-sensitive morphological changes, the polymer can undergo a reversible hydration-dehydration state transition near its lower critical solution temperature (about 32 °C). When this polymer is exposed to high temperatures, it dehydrates and then subsequently shrinks, reducing the spacing between the polymer particles within the HIO. Due to this transformation, a change within the diffraction spectrum occurs resulting in a visible colour change. The results obtained by Takeoka et al. indicated that the diffraction wavelength of the NIPA polymer varied from 780 to 350 nm when the temperature increased from 10 to 35 °C, almost covering the entire visible spectrum [23].

Within an experiment carried out by Zhao et al. [24], a clear and specific structural colour change was achieved with a dual responsive HIO in response to different temperatures and pHs. To produce the HIO, an opal template consisting of 270 nm SiO_2_ spheres with a face-centred cubic (fcc) structure was used with the hydrogel monomers dimethylaminoethyl methacrylate (DMAEMA) and spiropyran-methacrylate (SPMA) as the precursor solution to fill the interstitial spaces of the PCC template. The resulting HIO sensor showed a visible colour change from green to violet when the temperature increased from 30 to 50 °C.

#### 3.1.2. Detection of pH

**Figure 6:**
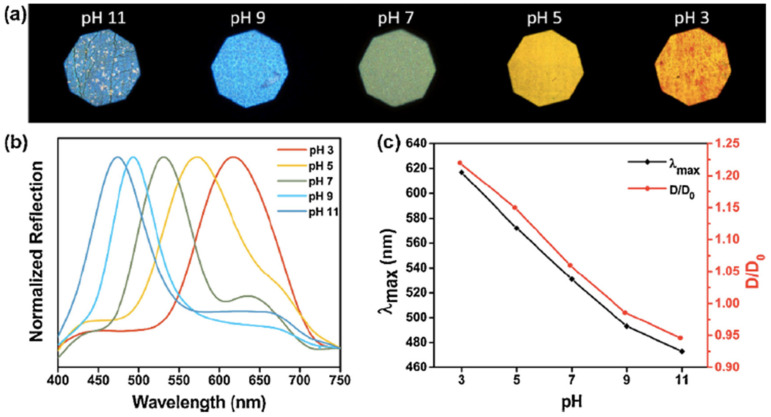
The pH responsive behaviour of the HIO sensor at different pH stimuli at a temperature of 30 °C. (**a**) photographs of the structural colour change. (**b**) The reflection spectra of the HIO sensor at five different pH’s (**c**) λ_max_ and the relative swelling ratio of the HIO at a temperature of 30 °C as a function of pH. Reprinted from Ref. [24]. Copyright 2018, Elsevier, Amsterdam, The Netherlands.

The detection of pH usually requires the use of IOHs [24,25,26,27]. A sensor produced by Zhao et al. [24] was designed to perform as a dual responsive HIO sensor, with structural colour change occurred at different pHs. Colours ranging from orange-red to yellow, dark green, indigo, and blue resulted when immersed in solutions of pH 3, 5, 7, 9, and 11, respectively, at a fixed temperature of 30 °C (see Figure 6), demonstrating a blue shift with increasing pH.

Another work on pH-responsive sensors were carried out by Yoon et al. [25]. Their method involved producing an inverse opal photonic gel (IOPG) with the addition of acrylic acid (AA) as a sensing monomer. With the addition of AA, distinct reflective colours were exhibited at different pHs due to changes within the refractive index. The AA-containing IOPG (AA-IOPG) had a green colour at a pH of 2, while a red-shift occurred when the pH was increased, due to an altered Donnan equilibrium by the deprotonation of AA. The pH-driven swelling of the AA-IOPG can be further investigated more precisely by obtaining reflectance measurements. Within this method, the maximum reflective peak shifted more than 100 nm as the pH increased from 2 to 7 [25].

A similar IOPG sensor was also developed by Park et al. [26] where once again AA was used as a moiety to make the polymer responsive to pH changes; however, this time to provide an ionizable carboxyl group. Park et al.’s sensor also underwent a colour change from green to red with increasing pH. In addition, their study involved tackling the problem of using benchtop equipment which is expensive and bulky by investigating a smartphone-based mobile method as a simple, inexpensive, and rapid alternative to obtaining data. Through rigorous experimentation the smartphone based analysis of the IOPG sensor was found to achieve reasonable precision under illuminations such as fluorescent light, LED, or the built-in flash of the smartphone [26].

#### 3.1.3. Detection of Humidity

**Figure 7:**
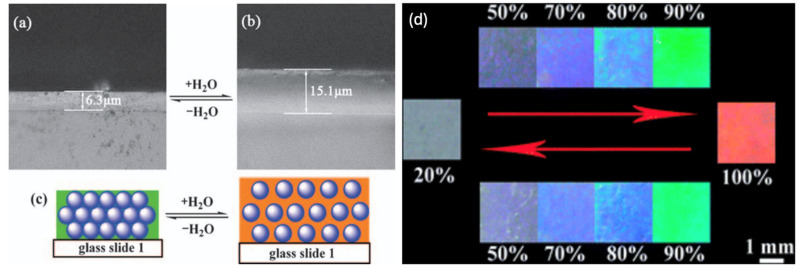
A PC-based sensor for detecting humidity: (**a**–**c**) alteration of PC particles within a hydrogel, (**d**) colours of the hydrogel at different humidity. Reprinted from Ref. [28]. Copyright 2008, Royal Society of Chemistry, London, UK.

Humidity monitoring is important within various environments. Industries such as the pharmaceutical, health and food industries require the constant monitoring of humidity to prevent problems such as mould growth from occurring. Humidity is also important to monitor within a laboratory as it can influence the reliability of results. Over the last few years, a large amount of effort has gone into producing humidity detection techniques. So far, however, conventional detection methods usually work by receiving electrical signals, and consequently a challenge to produce a simpler and more stable sensor remains [28]. Optical detection due to a colour change offers one of the best alternatives to detect a change in humidity without complex data collection and a display unit. Once again, polymer hydrogels can be used for this purpose [28,29,30]. Tian et al. [28], for instance, infiltrated a PCC template consisting of monodisperse latex spheres of poly (styrene-methyl methacrylate-acrylic acid [P(St-MMA-AA)) with a diameter of 150 nm with an acrylamide (Aam) solution to produce a PCC hydrogel as a humidity-responsive sensor. Figure 7a demonstrates that when the humidity within an environment rises, the PCC hydrogel absorbs water and swells, resulting in a red-shift of the material. In addition, this PCC hydrogel sensor can undergo a reversible colour change, as when the humidity decreases, the PCC hydrogel will return to its original state (blue structural colour). Figure 7b illustrates the reversible colour changes at humidity levels from 20 to 100%.

In another study carried out by Sobhanimatin et al. [29], a HIO humidity sensor was fabricated through the copolymerisation of acrylamide, 2-acrylamido-2-methylpropane sulfonic acid (AMPS), and N,N’-methylene-bis-acrylamide. This sensor had the ability to undergo a PBG red-shift of 88 nm from 427 nm (violet) to 514 nm (green) when the relative humidity was increased from 20 to 90%. Due to the addition of the AMPS, which acted as an ionic functional monomer, the sensitivity of the HIO film increased by about 57 nm in comparison to the HIO without AMPS. This HIO sensor also demonstrated a very short response time: 2 s for a relative humidity variation from 0 to 80%. Additionally, this sensor exhibited excellent mechanical stability, recoverability, and a lifetime of more than a year without sensible degradation [29].

#### 3.1.4. Detection of Mechanical Force

**Figure 8:**
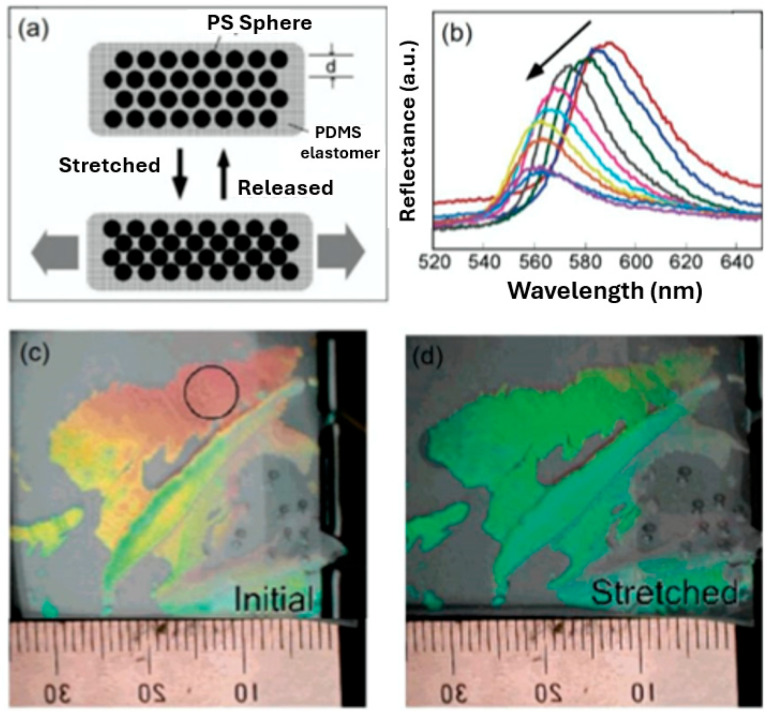
A PC hydrogel sensor for the detection of mechanical force. (**a**) The sensing mechanism, (**b**) Bragg diffraction reflectance of the HIO under stretching, and colour change of the film under (**c**) initial and (**d**) stretched conditions. Reprinted from Ref. [31]. Copyright 2006, American Chemical Society, Washington, DC, USA.

The detection of mechanical force is often required in areas where a structural change needs to be monitored, such as upon bridges and buildings. Due to the mechano-responsive properties of hydrogels, the capacity to effectively control a structural change can occur due to their ability to undergo a colour change in response to stretching. Mechano-responsive hydrogels are classified as materials which can adjust and adapt their physiochemical properties in response to applied mechanical force/deformation [18]. For instance, a PC hydrogel sensor was produced by Fudouzi et al. [31] by filling a 202 nm spherical PS PCC template with polydimethylsiloxane (PDMS). After swelling with silicone oil, a reversibly tuneable elastic-responsive PC hydrogel was achieved—see Figure 8. Figure 8a demonstrates that when the PC hydrogel is stretched horizontally, the size within the vertical direction is reduced, resulting in a decrease within the lattice spacing on the (111) crystal plane and inducing a blue shift within the material. Figure 8b shows that the diffraction peak blue-shifted from 590 to 560 nm, however with decreasing intensity, while Figure 8c,d demonstrate the visible colour change of the material before and after stretching.

#### 3.1.5. Detection of Environmental Pressure

PCCHs are a suitable material to serve as a sensor for environmental pressure [32,33]. When a gradient pressure is applied to a colloidal crystal film in a specific direction, a change within the periodic arrangement of the particles occurs. Studies carried out by Ding et al. [32] dispersed SiO_2_ microspheres within a pre-gel solution consisting of polyethylene glycol diacrylate, polyethylene glycol, and 2-hydroxy-2-methyl-1-phenyl. The microsphere-gel composition was then self-assembled within a rectangular mould and then cured with UV light to obtain an ordered colloidal crystal film. When a uniform pressure gradient is applied to the colloidal crystal film, the periodic arrangement is altered, initiating a colour change within the film [32].

Within another study carried out by Escudero et al. [33], a pressure sensor was developed using a 2D PCC template consisting of 800 nm polystyrene particles which was infiltrated with the elastomer polydimethylsiloxane (PDMS) and integrated with a microfluidic system. When different values of pneumatic pressure were applied, a change within the reflective colour was obtained and then evaluated with UV-Vis reflection spectroscopy. A maximum sensitivity of 0.17 kPa^−1^ was achieved with this sensor. To address the issue of traumatic brain injury (TBI) of soldiers who have been exposed to blast shockwaves and athletes exposed to concussion, Cho et al. [34] attempted to design a power-free, light-weight, wearable patch which could undergo an immediate colour change in response to impact pressure. To achieve this, an IO material was designed by infiltrating silica particles with diameters 320, 285, 238 nm with the uncross-linked thermoplastic polymer SU-8, then removing the silica template via chemical etching with hydrofluoric acid.

Table 1 summarizes hydrogel-based sensors that have been used for the detection of physical changes including temperature, pH, humidity and mechanical force.

### 3.2. IO and PCCH for Sensing Chemical Compounds

Chemically stimuli-responsive sensors have various applications. The detection of organic solvents, heavy metal ions, gases, organic gases, and toxic organic pollutants can all be detected using a variety of sensors. Since the beginning of the Industrial age within the mid-18th century, a high number and a variety of pollutants have been released into the environment. Harmful pollutants have been released into rivers, lakes, streams, and oceans causing a huge impact upon our ecosystems, wildlife, and human health. It is therefore of paramount importance that these pollutants are monitored to avoid the detrimental effects upon our environment.

#### 3.2.1. Detection of Alcohols

A rapid, sensitive, selective and quantitative detection of alcohols is required in law enforcement, forensics, clinical settings, chemical, pharmaceutical, and fermentation industries [35]. Within the wine industry, for instance, an efficient and rapid detection of ethanol detection below 20% is required for the production of quality wines [36]. The development of a sensor which can undergo a rapid structural colour change in response to ethanol is therefore required. This has been investigated and addressed within various studies with the use of PCC and IO-based materials [36,37,38,39]. Currently, there are three main structural designs for a colorimetric indicator of ethanol concentrations: opal, opal composite films, and inverse opal [36]. Within a study carried out by Rashidi et al. [37], silica IO was used for the detection of different concentrations of ethanol by determining the optical intensity of an absorbance peak. Their studies found that the intensity of the absorbance spectrum decreased with increasing concentrations of ethanol (from 2 to 100%), establishing a perfect linear relationship (R^2^ = 0.98). Liu et al. [38] produced PCC composite films composed of hollow silica microspheres which produced a λ_max_ span of ~50 nm responding to ethanol concentrations from 0 to 100%, while Qi et al. [39] constructed a shape memory IO colorimetric sensor with a λ_max_ span of ~140 nm for ethanol concentrations ranging from 0 to 100%. To achieve an observable structural colour change, a greater λ_max_ span is required. Additionally, it is necessary to detect ethanol concentrations from 0 to 20% to monitor the fermentation process. To solve this problem, Yuan et al. [36] produced a sensor consisting of a double inverse opal photonic crystal (DIOPC) which underwent a λ_max_ of up to 166 nm in response to ethanol concentrations from 0 to 100%. Additionally, this sensor exhibited a rapid response rate of <1.5 s while demonstrating structural colours from blue to red. The mechanism behind this sensor was due to the solvent-triggered recovery of the IO skeleton, the swelling/shrinking properties of the acrylamide polymer, the phase separation of the polystyrene (PS) microspheres and polymer skeleton, and the light diffraction of the DIOPCs [36]. This DIOPC sensor therefore has potential within the fermentation industry due to its ability to undergo a relatively large λ_max_ change [40].

#### 3.2.2. Detection of Volatile Organic Compounds and Organic Solvents

Organic solvents are used within the production of paints, lacquers, glues, cleaning agents, dyes, and agricultural products, and invariably enter our environment. Carcinogenic volatile organic compounds (VOCs) such as benzene, trichloroethylene, and carbon tetrachloride, or neurotoxins such as n-hexane, toluene and tetrachloroethylene are therefore obviously a concern and need to be monitored. Due to these concerns, investigation and research has gone into the development of sensors to detect these pollutants. Various methods have been investigated using silica and metal oxide IO, and PCCHs [41,42,43,44,45]. For instance, in 2007, Endo et al. [41] prepared a PCC-based sensor upon a glass surface that was able to produce a visible structural colour change in response to various VOCs including acetone, toluene, benzene, and xylene. This was achieved by filling a PS (particle diameter of 202 nm) PCC array with the elastomer polydimethylsiloxane (PDMS), which was infiltrated into the PCC through capillary action [41]. Due to the swelling properties of this matrix with the addition of acetone, or a benzene, toluene, xylene (BTX) mixture, a structural colour change was observed with the naked eye [41]. Another study carried out by Zhang et al. [42] produced a SiO_2_ IO sensor for the detection of volatile organic solvents. Within their experiment, they found that by infiltrating tetraphenylethene polymer (TPEP) into the voids of the SiO_2_ IO, the PBG could be greatly enhanced for the detection of VOCs in comparison to SiO_2_ IO without TREP. This TREP-functionalised IO material has the capacity to detect (VOCs) such as tetrahydrofuran (THF), acetone, benzene, and toluene with increased sensitivity. When THF reacts, or is absorbed by the TREP molecules, it causes the refractive index change of the PCC structure, resulting in an observable structural colour change. The PBG change of the as-prepared sensor when exposed to saturated THF vapor shifted from the initial 450 nm to 512 nm (a red-shift of 62 nm), while when exposed to acetone vapor, a red shift of around 50 nm occurred. The increase of the PBG shift can be attributed to the strong adsorption of the analytes to the TPEP molecules and demonstrates one way the sensitivity of an IOPC can be improved.

Within a study carried out by Schroden et al. [43], a zirconia (ZrO_2_) IO structure was produced from a PCC template consisting of different sized PS particles (200, 250 and 285 nm) for the detection of methanol. Their studies not only demonstrated that a zirconia IO structure is an efficient material for the detection of methanol, but also how the pore size of an IO structure influences the structural colour. As mentioned earlier, the pore size of an IOPC is an important consideration regarding the color produced by the structure. Larger pore sizes will produce colors at the red end of the spectrum, while smaller pore sizes towards the violet end of the spectrum.

Lu et al. [44] combined and exploited the properties of both IO and chromophores to enhance the sensitivity, selectivity, and reusability of a sensor for the detection of formaldehyde (FA) and acetaldehyde (AcH) within air, aquatic products, and cells. This was achieved by infiltrating a silica IO structure with a naphthalimide derivative fluorophore. This sensing system utilised the slow photon effect of the IO, and the fluorescent properties of a naphthalimide derivative to improve both the response speed and the detection sensitivity, producing more intense colours at around 550 nm. Additionally, this as-prepared IO sensor has the advantage of being reused simply by washing it within an aqueous acidic solution.

**Figure 9:**
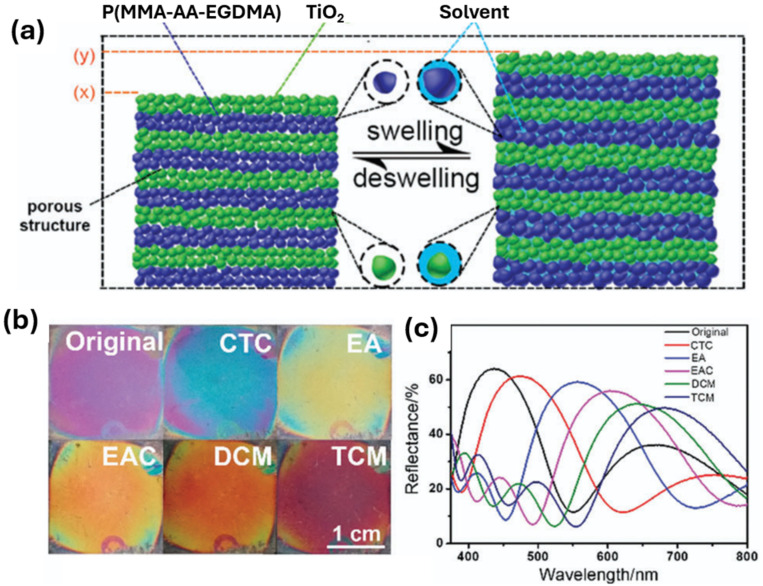
(**a**) The PCC produced by the layer-by-layer spin coating method (left), before and after being placed in an organic solvent. (**b**) Photographs of the 1D PCC sensor in different solvents, (**c**) The reflectance spectra of the sensor within different solvents. Reprinted from Ref. [45]. Copyright 2018, Royal Society of Chemistry, London, UK.

In another study, Kou et al. produced a porous organic/inorganic hybrid 1D PCC structure via the layer-by-layer spin coating method, consisting of alternating layers of poly(methyl methacrylate-acrylic acid-ethyleneglycol dimethacrylate) (P(MMA-AA-EGDMA)) nanoparticles and a suspension of TiO_2_ nanoparticles on a silicon substrate for the detection of VOCs [45]. By altering the fabrication parameters such as the spin rotation speed, spin coating times, the stack number, and the concentration of TiO_2_, different layer thicknesses of the 1D PCC could be achieved. Kou et al. experimented with the optical properties associated with these different parameters for the detection of various VOCs such as carbon tetrachloride (CTC), ethyl acetate (EAC), ethanol (EA), chloroform (TCM), and methylene chloride (DCM). It was discovered that by altering the layer thickness, different structural colours can be achieved. For instance, when the TiO_2_ concentration was kept constant, but the spin rotation speed was increased from 3000 to 4000, and then 5000 rpm, the layer thickness gradually decreased, leading to a colour change from blue-green to dark blue, and then to purple, respectively. In addition, the spin coating times also influenced the thickness. It was discovered that when the spin rotation speed was kept constant at 4000 rpm, and the spin-coating times were increased from twice, to three times, then to four times, the colours changed from a lavender to dark violet, then to blue colour. After experimenting with synthesis parameters, a 3-stack 1D PCC was developed by Kou et al. by alternatively spin-coating TiO_2_ solution twice, and the microemulsion twice at 3000 rpm [45]. The material was then tested in response to CTC, EA, EAC, DCM, and TCM and was discovered that the colours changed quickly within 2 s from violet to blue, yellow, orange, orange-red and then black-red, respectively, as shown in Figure 9b, while the maximum reflectance wavelengths shifted from the original 437 (1D PCC without a VOC) to 471, 556, 601, 645 and 682 nm, respectively, as shown in Figure 9c.

#### 3.2.3. Organic Pollutants

Organic pollutants are a major concern worldwide and enter into the world via agricultural contaminants and industrial sectors which then find their way into the food chain. Exposure to these pollutants have the ability to cause various health problems: cardiovascular diseases, endocrine disruption, cancers, birth defects, diabetes, and dysfunctional immune and reproductive systems [46]. It is therefore of paramount importance that a fast and effective way to detect these pollutants is developed. Some of the main pesticides currently used in agriculture include atrazine, glyphosate, and 2,4-D [47]. Methanephosphonic acid (MPA), for instance, is a breakdown product of glyphosate which can impose minor health problems. To address this, Huang et al. [48] developed a method using molecularly imprinted technology (MIT) combined with HIO. MIT technology [49,50] involves producing a polymetric matrix which is able to mimic natural recognition entities such as antibodies, biological receptors [50], or other molecules [49]. With the combination of MIT and HIO, particles were produced with a diameter range from 300 to 635 μm and infiltrated with MPA. Within their studies, Huang et al. found that even at small amounts of MPA (10^−6^ M) induced a large red-shift of the PBG which was recognised with the naked eye. The results also showed that the limit of detection (LOD) went from 1 × 10^−6^–1 × 10^−3^ M to 1 × 10^−6^ to 5 × 10^−3^ M as the IOH particles enlarged from 300 to 635 μm [48], demonstrating that the sensitivity of the MPA detection decreased according to an increase in the size of the particles. Their study showed that the IOH MPA-imprinted particles provided a good strategy for a highly sensitive colorimetric sensor for phosphorous-based pesticide detection, and consequently has potential applications in food safety, eco-pollution determination, and nerve gas detection [48].

In an experiment carried out by Li et al. [51], the detection of the organic pollutant p-nitrophenol (PNP) was performed. The monitoring of this pollutant is important as it undergoes bioaccumulation and biomagnification, is very carcinogenic, and tends to persist in water and soil [52]. Within their study, an observable structural colour change was achieved using an HIO material composed of the polymers hydroxyethyl methacrylate (HEMA) as a functional monomer, and ethylene glycol dimethylacrylate (EGDMA) as a crosslinker. It was discovered that using HEMA as a functional monomer, in comparison to other functional monomers such as methacrylic acid, acrylic acid, or acrylonitrile, the response performance was much greater. Their experiment discovered that by altering the ratios of HEMA and EGDMA, the Bragg diffraction peak was influenced. When the molar ratios between the HEMA and EGDMA were at a ratio of 5:1, the greatest red-shifted occurred at 51 nm. This ratio enabled the sensor to be more sensitive and selective for the detection of PNP where a visible colour change was produced from green to red with a concentration from 0 to 80 mM [51].

#### 3.2.4. Detection of Heavy Metal Ions

**Figure 10:**
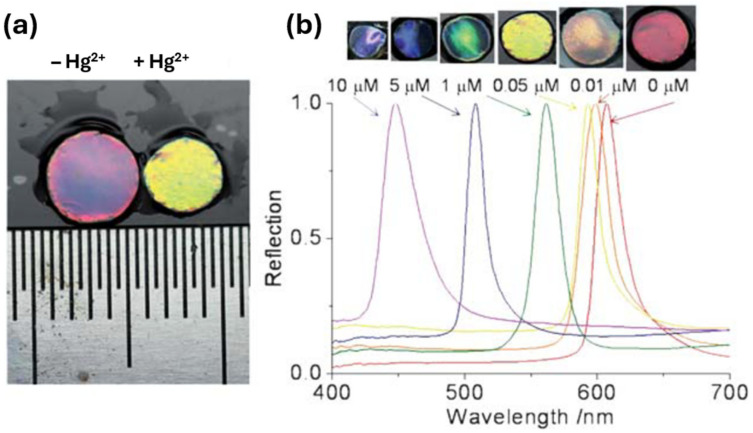
(**a**) Volume change of the PCCH sensor after binding with Hg^2+^. (**b**) Effect of the Hg^2+^ concentration on the color (top) and diffraction wavelength (bottom) of the sensor. Reprinted from Ref. [53]. Copyright 2012, Royal Society of Chemistry, London, UK.

Heavy metal ions are a problem within the environment due to their high toxicity, wide pollution range, and bioaccumulation, posing a serious threat to human health and the ecological balance [3]. The detection of heavy metals is therefore required to monitor and eliminate their presence within the environment. Once again, as with the monitoring of many chemicals within the environment, there is a need for low-cost, easy-to-use sensors that offer high sensitivity and real-time detection. PCC sensors for heavy metal detection involve the use of PCCH, HIO, and silica and chitosan-based IO structures [53,54,55,56,57]. For example, Ye et al. [53] developed a method of producing a PCCH whereby monodisperse silica nanoparticles were polymerized within a polyacrylamide hydrogel for the detection of Hg^2+^ and Pb^2+^. This was achieved by using ion-responsive aptamers which were crosslinked within the hydrogel network for the detection of these heavy metals. A structural colour change was observed during the detection of Hg^2+^ and Pb^2+^ due to the specific binding of the and cross-linked single stranded aptamers which caused the hydrogel network to shrink, producing a blue shift within the Bragg diffraction peak (see Figure 10).

As can be seen in Figure 10, a large volume change occurred due to the binding of the aptamer to Hg^2+^ producing a blue shift. The concentration of the Hg^2+^ (Figure 10b) demonstrates the volume change in accordance with the concentration of Hg^2+^. The degree of the blue shift within the Bragg diffraction peak therefore could be used to estimate the quantity of the target ion. This sensor displays a high sensitivity and observable colour change within the material and has the potential to be used for the screening of a wide range of heavy metal ions within food, drugs, and the environment [53].

In another study, Zhang et al. [54] carried out an experiment using carboxylated HIO as a sensing platform for the detection of Hg^2+^. Their experiment consisted of using HIO to determine a diffraction wavelength shift when immersed within a Hg^2+^ aqueous solution. A blueshift was observed due to the shrinkage of the HIO structure resulting from the formation of metal complexes with the Hg^2+^ ions and the carboxylate anion. Other methods to detect heavy metal ions have employed the use of silica-based and metal oxide IO. For instance, studies carried out by As’adi Harab et al. [55] used silica IO to detect 20 different metal salts. Due to the competition between the hydrophilicity of the SiO_2_ and the hygroscopicity of the metal salts, a PBG shift towards the blue or red end occurred due to the shrinkage or expansion of the IO pores. It was found that a blueshift occurred when the silica IO lost trapped water molecules within its pores resulting in a shrinkage. Conversely, when silica absorbs more water molecules, the IO pores expand resulting in a redshift. The major PBG shift occurred during the addition of the mercury (II) nitrate salt. With a concentration of 0.002 mol/L, a 19 nm shift towards the red end of the spectrum occurred, which is equivalent to a sensitivity of 9.5 nm/mM, while the minimum PBG shift occurred with the sodium nitrate salt which underwent a 5 nm shift towards the blue end of the spectrum, which is equivalent of a sensitivity of 2.5 nm/mM [55]. In another study carried out by Li et al. [56], silica IO was used to detect the presence of Hg^2+^ ions in aqueous medium. Within their experiment, the fluorescent probe tetraphenylporphyrin (TPP) was used to increase the intensity during the detection process. This was performed by adding 50 μL of a TPP–chloroform solution onto the surface of silica IO, resulting in a fluorescent signal amplified up to 38.9-fold compared to that without. This method was determined to have a low LOD of 1.18 × 10^−9^ mol/L.

Another method of detecting heavy metal ions, carried out by Su et al. [57], involved the use of chitosan-based IO particles. Chitosan’s fluorescent properties can undergo a decrease in fluorescence intensity when it encounters the heavy metal ion hexavalent Cr(VI), and consequently these particles can be utilised to detect Cr(VI) within drinking water samples. Within their assay, a fluorescence microscope was used to determine the intensity before and after the addition of Na_2_CrO_4_ solution. This method demonstrated a high sensitivity with a LOD of 0.055 μM.

#### 3.2.5. Detection of Gases

The detection of gases is an important requirement within various fields such as the oil industry, mining, medical devices, wastewater, power stations, etc. Gas sensors are therefore of great importance. At present, various methods to detect organic gases include high-performance liquid chromatography (HPLC), gas chromatography-mass-spectroscopy (GC-MS), surface enhanced resonance Raman scattering (SERRS), quartz crystal microbalance (QCM) [58], and conductometric gas sensors [59]. These methods, however, are expensive, time consuming, and require trained personnel to operate them. HIO, silica, and metal oxide IO sensors have therefore been investigated to tackle these problems [58,60,61]. Inverse opal structures are preferred for gas detection due to their high surface area, which is often larger than that of opal structures [3]. The inter-connected pores within the inverse opal can improve the diffusion rate of gas molecules within the material and consequently improve the response speed and sensitivity of the PCC sensor [3]. For instance, Heo et al. [60] developed the real-time monitoring of CO_2_ gas using a HIO consisting of 2-(dimethylamino)ethyl methacrylate (DMAEMA). This sensor produced a visible structural colour change due to the swelling properties of the HIO in response to CO_2_. As the CO_2_ concentration varied from 0 to 100% a structural colour change from green to red was observed, making it an efficient colorimetric sensor for the detection of different CO_2_ concentrations.

In a separate work, Zhang et al. [58] fabricated a SiO_2_ IO sensor infiltrated with Fluoral-p for the detection of FA vapour. When this sensor encountered FA vapour, a fluorescence signal was produced within 20 s, due to the reaction between Fluoral-p and FA. In addition, the sensor produced by Zhang et al. had a high sensitivity with a LOD of 0.008 mg m^−3^, which is 10 times lower than the maximum safe concentration for indoor air permitted by the World Health Organisation (WHO) [58]. Within other studies, the detection of acetone and formaldehyde vapours was successfully achieved by Chen et al. [61] using a metal oxide IO consisting of SnO_2_ doped with cobalt. It was reported that by doping the SnO_2_ with cobalt, the Fermi level position was elevated, the PBG was decreased, and an increase of the chemisorbed oxygen was achieved, resulting in the enhancement of the sensing performance to formaldehyde and acetone vapour.

Table 2 summarizes sensors that have been used for the detection of chemical substances.

### 3.3. Applications of IO as Biosensors

#### 3.3.1. Detection of Biomarkers

The detection of biomarkers is an important and effective way of diagnosing diseases, and specific diseases have particular biomarkers associated with them. Early diagnosis is therefore important to prevent the onset of diseases. For instance, the biomarker D-dimer is associated with the venous thromboembolism (VTE) disorder, which is a leading cause of death worldwide [62]. To detect this biomarker, Pereira et al. [62] developed a molecularly imprinted polymer (MIP) HIO, which they termed a photonic molecularly imprinted polymer (PMIP). Sensors based upon MIPs are an appealing way of detecting biomarkers where the MIP is assembled onto photonic crystals [62]. Within Pereira et al.’s experiment, the D-dimer showed a very low LOD ranging from 22.5 to 1450.0 ng mL^−1^.

Extracellular vesicles (EVs) are nanosized assemblies contained by a lipid bilayer membrane which are released from cells as part of their routine processing. EVs contain specific biomolecular content in the form of proteins, DNA, RNA, and lipids which are essential for intercellular communication and disease propagation [63]. EVs are therefore promising candidates for diagnosis within neurology, cardiology, and oncology.

Studies using silica and metal oxide IO have been used to detect EVs [63,64]. For instance, in an experiment carried out by Suthar et al. [63]. quartz crystal microbalance technology was used in conjunction with an antibody-functionalised silica IO film for the immunocapture of the CD63-positive EV. The CD63-positive EV occurs at high levels in patients with pancreatic cancer and is therefore an important diagnostic biomarker [65]. Suthar et al.’s experiment discovered that this method provided a LOD as low as 6.24 × 10^7^ particles per mL [63]. Within a study carried out by Dong et al. [64], Au-coated TiO_2_ macroporous IO was used to capture EVs and obtain specific spectroscopic information using surface-enhanced Raman scattering. Due to the ability of the TiO_2_ IO to capture the exosomes from the plasma of cancer patients, it was discovered that there was a SERS intensity of 1087 cm^−1^ resulting from the P-O bond within the phosphoproteins within the exosomes [64]. This intensity reading was found to be around two times greater than that of healthy people. This method therefore proved to be a simple and versatile method for cancer diagnostics.

#### 3.3.2. Detection of Tumour Cells

**Figure 11:**
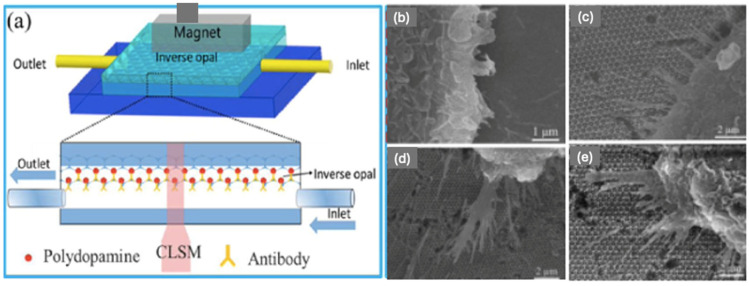
(**a**) Schematic diagram of the IO based on microfluidic chip. SEM Images of captured cancer cells on (**b**) flat glass, (**c**) pore size 250 nm, (**d**) pore size 305 nm, (**e**) pore size 415 nm IO structures Reprinted from Ref. [66]. Copyright 2017, American Chemical Society, Washington, DC, USA.

A sensitive detection and screening method of circulating tumour cells (CTCs) has evoked a lot of attention over the last couple of decades due to the demand for prognosis and the selection of an appropriate treatment. This requirement, however, has remained technically challenging due to the extremely low abundance of CTCs within a large number of hematologic cells in the blood [66]. A more efficient method is therefore required. Xu et al. [66] developed a 3D IOPC-based microfluidic chip for the capture of CTCs combined with Fe_3_O_4_@C6@silane nanoparticles (see Figure 11a). The antibody Anti-EpCAM was conjugated onto the interface of the IOPC with polydopamine for the recognition and detection of CTCs. Anti-EpCAM antibodies are immunodetection reagents used for the detection of a target antigen, epithelial cell adhesion molecule. The protein is encoded by the EpCAM gene in humans.

As illustrated in Figure 11a, the IO substrate served as a capture interface. The chamber consisted of the IO modified with the anti-EpCAM. When the magnetic-labelled CTCs were pumped into the chamber, the tumour cells were pulled along by the magnetic field and came into contact with the IO structure and consequently be captured by the anti-EpCAM antibodies. The study found that the CTC capture efficiency was dependent on the pore size of the IO substrate. The IO structure-based microfluidic chip was constructed with the pore sizes of 250, 305, and 415 nm, see Figure 11c–e, where the corresponding cell capture efficiencies were 38.4%, 50.56%, and 85%, respectively [66].

#### 3.3.3. Detection of Carcinogenic Mycotoxin Ochratoxin A

Within a study carried out by Cui et al. [67], the successful detection of the carcinogenic mycotoxin Ochratoxin A (OTA) was achieved using a HIO film consisting of the polymer poly (ethylene glycol) diacrylate (PEGDA) covalently coupled with aptamers to selectively recognise OTA. To address the issue of the need to perform on-site detection without complex laboratory procedures, a portable microscope and smartphone was used for the quantitative detection of OTA. This was achieved by developing a written algorithm for a smartphone and then using the camera to capture colour changes upon the HIO film from a portable microscope. It was reported that this portable platform achieved a LOD of 1 μg/mL without the need for bulky and expensive equipment.

#### 3.3.4. Detection of the Influenza Virus Using IO

**Figure 12:**
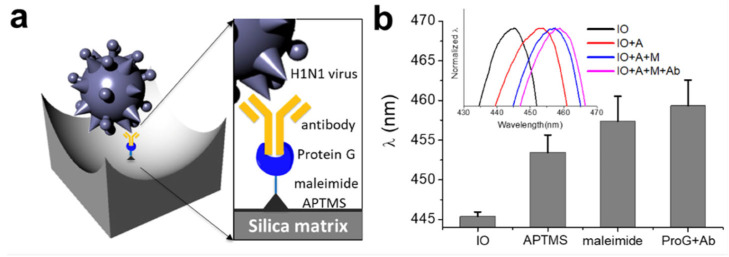
(**a**) Illustration displaying the molecular structures formed by the functionalisation upon the IO structure for binding the H1N1 subtype; (**b**) Reflectance peak positions for the pristine IO, APTMS-treated, NHS-PEG4-Malemide cross linker-treated, and Cys-ProG-antibody immobilized IOs. Inset shows reflectance spectra for all four samples. APTMS: 3-aminopropyltrimethoxysilane. Reprinted from Ref. [68]. Copyright 2018, MDPI, Basel, Switzerland.

Influenza is an acute infectious disease caused by the influenza virus belonging to the *Orthomyxoviridae* family, which is divided into three types: influenza A, B, and C [68]. The fourth virus from the *Orthomyxoviridae* family is influenza virus D, which only affects animals with little known on its impact upon human health [69]. As it is a serious public health threat, a rapid diagnosis is required to prevent the spread of the disease. To address this problem, Lee et al. [68] developed an IO structure consisting of SiO_2_ for the immobilisation of the influenza virus antibody using chemical and biological linkers (see Figure 12). It was discovered that this SiO_2_-IO system successfully immobilised the H1N1 subtype when immersed within a dispersion of the viruses, leading to a red shift of the reflectance peak. Furthermore, it was found that the H1N1 was sensitively and selectively detected within a concentration range from 10^3^ to 10^5^ Plaque Forming Units (PFU).

#### 3.3.5. Detection of Amino Acids

**Figure 13:**
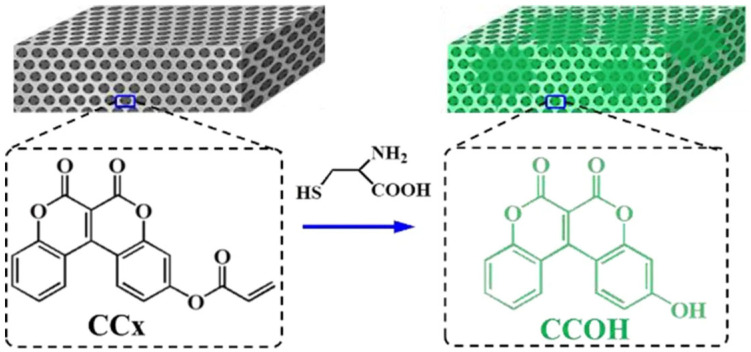
An illustration of the detection mechanism for the CCx IO films. The organic molecule CCx is adsorbed onto the microporous wall of the IO which reacts with cysteine to produce the fluorescent product CCOH when the CCx IO film is exposed to cysteine solution. Reprinted from Ref. [70]. Copyright 2023, Springer, Berlin/Heidelberg, Germany.

The amino acids such as cysteine (Cys) play a vital role in various processes such as enzymatic reactions and heavy metal resistance. Abnormal levels of this amino acid are associated with slow growth, cancers, liver damage, and Alzheimer’s and Parkinson’s diseases [70]. It is therefore important to develop a simple, convenient, and highly sensitive method to detect this amino acid. A study carried out by Li et al. infiltrated an IO structure consisting of SiO_2_ with a coumarin derivative, 6,7-dioxo-6H,7Hchromeno [3,4-c] chromen-3-yl acrylate (denoted as CCx) to produce a fluorescent sensing film for the detection of the amino acid cysteine within human serum and living cells [70]. This was achieved by the CCx reacting with cysteine to produce the fluorescent product 3-hydroxy-6H, 7H-chromeno [3,4-c] chromene-6, 7-dione (denoted as CCOH). As demonstrated in Figure 13, the IO structure produced a fluorescent colour change when it encountered the amino acid cysteine. In addition, the intensity of the fluorescent product could be increased or magnified by the slow photon effect of the IO film.

#### 3.3.6. Detection of Bacteria

Bacteremia caused by bacterial bloodstream infections, such as *Staphylococcus aureus*, *Escherichia coli*, *Pseudomonas aeruginosa*, and so on can lead to health problems such as vascular leakage, tissue damage and multiorgan failure, accounting for one third of global mortality. For the clinical diagnosis of bacteremia, it usually takes around 3–5 days of incubation and 12 h of growing time to identify the bacteria [71]. Methods such as polymerase chain reaction, surface enhanced Raman scattering, and fluorescent probes require long-term blood culture and expensive equipment. Due to these problems, a faster diagnosis is in demand. To address this issue, Xu et al. [71] developed a technique using new barcode (also called encoded microcarriers or IO microspheres) technology, which can simultaneously capture and detect various types of pathogenic bacteria. These barcodes consist of a poly (ethylene glycol) (PEG) HIO synthesised using a SiO_2_ PCC template consisting of six kinds of nanoparticles with sizes of 211, 260 and 307 nm. The assembled templates were then immersed into a pre-gel solution consisting of 20% poly(ethylene-glycol) diacrylate (PEG-DA), 10% acrylic acid (AA) (PEG-DA), and 1% HMPP for 1 h. After removing the silica template using 4% hydrofluoric acid, the magnetic IO barcodes were saturated with magnetic nanoparticles.

**Figure 14:**
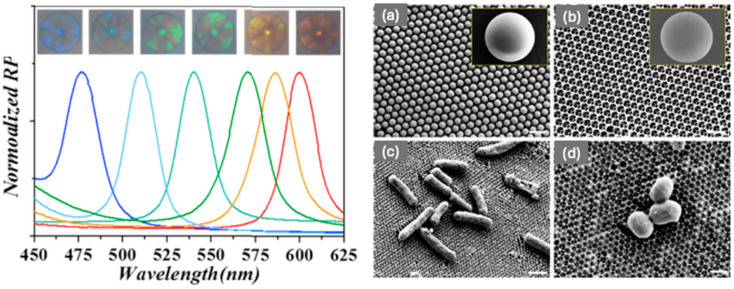
(**left**) Reflection spectra and optical images of the six kinds of hydrogel barcodes. SEM Images: (**a**) surface image of the SiO_2_ PCC template, (**b**) inverse opal barcodes, (**c**) the captured *E. coli* on the surface of the Apt-decorated IO barcodes, (**d**) the capture of the *S. aureus* on the surface of the Apt-decorated IO barcodes. The scale bars are 500 nm in (**a**,**b**), 2 μm in (**c**) and 500 nm in (**d**). Reprinted from Ref. [71]. Copyright 2018, Elsevier, Amsterdam, The Netherlands.

The IO barcodes were then functionalised with two kinds of aptamer probes (Apt*_S_._aureus_* and Apt*_E_._coli_*). The red IO barcodes were coated with the amino-modified Apt*_S_._aureus_* and the green IO barcodes were coated with the amino-modified Apt*_E_._coli_*. The microstructures of the IO magnetic hydrogel barcodes were observed with SEM. Figure 14a shows the silica nanoparticle template formed the hexagonal close-packed structure, while Figure 14b shows a typical IO structure was achieved. The SEM images from Figure 14c,d demonstrate and confirm the successful capture of *E. coli* and *S. aureus* onto the surface of the HIO barcodes. Within the experiment carried out by Xu et al., the use of the magnetic HIO barcodes to capture and identify *E. coli* and *S. aureus* could be achieved at low bacterial concentrations (100 CFU mL^−1^) within 2.5 h. This is a reduced time in respect to other methods used within a clinic [71].

#### 3.3.7. Detection of Biomacromolecules

**Figure 15:**
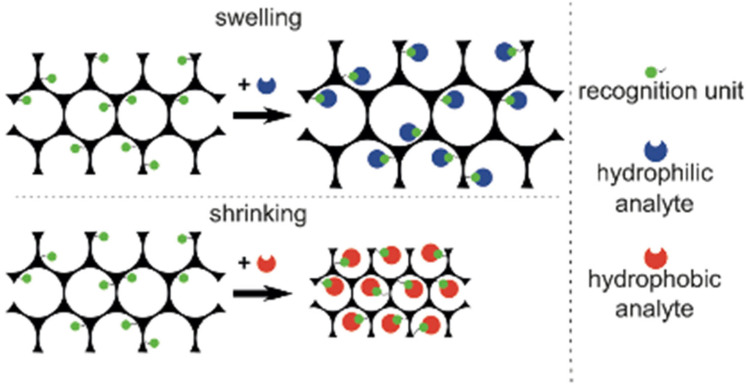
Volume changes of an HIO in response to a hydrophilic and hydrophobic analyte. Reprinted from Ref. [72]. Copyright 2015, Wiley, Hoboken, NJ, USA.

In the case of bioassays, HIOs which swell or shrink in response to changes of the hydrophilicity of the system are the most favourable methods used due to the binding of an analyte. Figure 15 demonstrates the swelling/shrinking properties of an HIO in response to a hydrophilic and hydrophobic analyte [72].

These sensors provide an easy, simple method of visual detection without the need for sophisticated equipment and trained operators. Consequently, HIO sensors have been investigated for the detection of low-molar-mass analytes. Sensing systems for the detection of large analytes, however, such as bio(macromolecules) including viruses, bacteria, and other macromolecules, have been largely unexplored. Within the few reported cases, the maximal shifts of the interference maximum were small, hardly exceeding 20 nm [72]. This was presumably due to two problems: firstly, a limited change of the hydrophilicity of the HIO upon binding of the analyte, and secondly, the problem of the diffusion of the bio(macromolecule) into the HIO, which is required to produce a change within the periodic structure and thus produce a visible structural colour change. To overcome these restrictions, Couturier et al. [72] produced a dual-responsive HIO which could not only facilitate an analyte through large channels but also undergo a volume phase transition to produce a strong signal. This was achieved by an HIO based on two different monomers of the copolymer oligo (ethylene glycol) for the sensing of bio(macromolecules). These copolymers are both biocompatible and display low-fouling behaviour, while also exhibiting a lower critical solution temperature (LCST) phase transition in aqueous media, which can be tuned by the copolymer composition.

In addition, the copolymers could easily incorporate additional functional comonomers serving as recognition units for the binding of specific analytes. If the binding of an analyte influences the overall hydrophilicity, the LCST will be shifted. This dual system therefore allows an induced phase transition under isothermal conditions if the binding of the analyte shifts the phase transition temperature from below to above the sensing temperature, or vice versa. This dual system therefore has the capacity to undergo greater volume changes within a particular chosen temperature window due to the alteration of the hydrophilicity of the HIO when the binding of an analyte occurs.

The operation principles and detailed detection of these biosensors are summarized in Table 3.

## 4. Conclusions and Outlook

Overall, PCCs have unique structural and optical characteristics that allow precise manipulation of light. PCCs can be utilised as sensors due to their ability to undergo a structural colour change, or measurable shifts in PBG in response to various forms of physical, chemical or biological environments. Within this review, the fundamental structure and optical properties of PCCs, and how they manipulate light is first discussed, followed by the different preparation methods of PCCs and IO. The application of these structures as sensors is then outlined for the detection of various forms of external stimuli, along with strategies to enhance their sensitivity and lower their LOD. In general, due to the optical properties imparted by PBGs and the versatility of IO structures derived from PCC templates, these sensors have found applications across various fields such as environmental monitoring, biomedicine, food safety, clinical diagnosis, and others. However, several challenges remain, such as minimizing defects during PCC fabrication, enabling sensor reusability, and achieving scalable production with improved thermal and mechanical stability.

Compared to conventional sensing methods such as electrochemical, SPR, and fluorescence techniques, IO-based sensors offer distinct advantages including label-free detection and direct visual readouts. Nonetheless, traditional methods currently provide higher sensitivity and have been successfully integrated into commercial devices, such as electrochemical glucose monitors. Encouraging progress, however, has been made in label-free sensing, exemplified by innovations like the hydrogel inverse opal (HIO) contact lens, which changes colour in response to elevated glucose levels. With continued advancements in material science and nanotechnology, PCC and IO-based sensing platforms are poised to evolve into more sensitive, selective and user-friendly point-of-care devices, capable of detecting a wide array of analytes without the need for complex laboratory instrumentation.

## Figures and Tables

**Table 1:** Examples of Physical Based Sensors.

Sensors	Range of Application	Advantages	Features	Operation Principles	Ref.
HIO with *N*-isopropylacrylamide (NIPA).	Temperature	Reversible hydration-dehydration	Visual colour change	Thermo-sensitive morphological changes	[23] Takeoka
HIO with dimethylaminoethyl methacrylate (DMAEMA) and spiropyran-methacrylate (SPMA)	Temperature and pH	Dual Sensor	Visual colour change	Thermo-sensitive morphological changes	[24] Zhao
Inverse opal photonic gel (IOPG)	pH	Large PBG shift—more than 100 nm from pH 2–7	Visible colour change	Red shift occurred due to deprotonation of Acrylic acid (AA)	[25] Yoon
HIO	Humidity	Large PBG shift—427–514 nm from 20–90% humidity.	Visible colour change	Swelling/ Deswelling	[29] Sobhanamatin
PC Hydrogel with polydimethylsiloxane (PDMS)	Mechanical Force	Reversibly tuneable elastic-responsive PC hydrogel	Visible colour change	Morphological change due to stretching/change in lattice spacing	[31] Fudouzi

**Table 2:** Summary of Chemical-Based Sensors.

Method/Sensor	Range of Application	Advantages	Features	Operation Principles	Ref.
Silica IO	Ethanol	Perfect linear relationship between optical intensity change peak and ethanol concentration	PBG change (45 nm) for conc. 0 to 100%	Refractive Index change	[37] Rashidi
PCCH with polydimethylsiloxane (PDMS)	(VOCs) acetone, toluene, benzene	Reversibly tuneable structural colour	Visible colour change	Swelling/ Deswelling	[41] Endo
Silica IO	Volatile organic solvents	Increased sensitivity due to TPEP	Silica IO filled with tetraphenylethene (TPEP) enhances PBG change	Refractive Index change	[42] Zhang
Zirconia IO	Methanol	Strong visible colour change	Different pore sizes produced different colours	Refractive index change	[43] Schroden
PCCH composed of silica nanoparticles polymerised within a polyacrylamide hydrogel	Detection of Hg^2+^ and Pb^2+^	Method has the potential to detect a wide variety of metal ions.	Volume changes due to binding of aptamers to Hg^2+^	HIO shrinkage producing a visible blue shift.	[53] Ye

**Table 3:** Summary of IO-Based Biosensors.

Method/Sensor	Range of Application	Advantages	Features	Operation Principles	Ref.
Molecular imprinted HIO poly (methyl methacrylate)	Detection of Biomarkers	Low LOD: 15.5 ng mL^−1^	Does not rely upon the use of antibody labelling	Swelling of the HIO polymer/change in the RI	Pereira [62]
Silica IO	Detection of biomarker CD63 extracellular vesicles (EVs)	Low LOD: 6.24 × 10^7^ particles per mL	Functionalised with antibodies	Quartz crystal microbalance	Suthar [63]
IOPC based microfluidic chip combined with Fe_3_O_4_@C6@silane nanoparticles	Detection of circulating tumour cells (CTCs)	High cell capture efficiency	Uses magnetically labelled CTCs	Antibody Anti-EpCAM was conjugated onto the interface of the IOPC	Xu [66]
HIO film consisting of the polymer poly (ethylene glycol) diacrylate (PEGDA)	Detection of mycotoxin Ochratoxin A (OTA)	A portable smartphone was used to capture colour changes	Uses aptamers to selectively recognise OTAs	Swelling/ Deswelling	[67] Cui
Silica IO	Detection of cysteine	Sensitive, selective, and fast response to cysteine	Can detect cysteine in human serum and living cells	Fluorescence colour change	[70] Li
Poly (ethylene glycol) (PEG) HIO spherical particles	Detection of bacteria	Low LOD: captured bacteria conc. 100 colony forming units (CFU) mL^−1^	Decorated aptamer probes within the PEG HIO	Fluorescence Intensity changes	[71] Xu
